# Online event on 19 November 2021: *Eurosurveillance* 25th anniversary seminar ‘Epidemiology and immunology – from observation to explanation and public health action’

**DOI:** 10.2807/1560-7917.ES.2021.26.44.211104m

**Published:** 2021-11-04

**Authors:** Eurosurveillance editorial team

**Affiliations:** 1 European Centre for Disease Prevention and Control (ECDC), Stockholm, Sweden

As part of its 25th anniversary celebrations, *Eurosurveillance* is hosting a scientific seminar on 19 November 2021 entitled ‘Epidemiology and immunology – from observation to explanation and public health action’.

On many occasions, epidemiologists’ observations of various vaccine preventable diseases patterns have generated hypotheses about potentially underlying immune mechanisms that support or interfere negatively with vaccination efforts. They have collaborated with virologists and immunologists, who through targeted research have been able to shed further light on the causing phenomena. Translation of this knowledge into practice has supported the development and further study of interventions to prevent infections and control the spread of disease. The ongoing COVID-19 pandemic continues to illustrate the important roles of different disciplines in understanding transmission patterns and pathomechanisms to support public health action. 

The seminar will present examples of the interplay between generating hypotheses from observation, researching associated virological and immunological mechanisms, and translating the knowledge gained into public health interventions. It will cover influenza, measles and COVID-19 and present epidemiological, virological/immunological and public health policy perspectives.

Speakers at the event moderated by Daniel Lévy-Bruhl, Santé publique France, are:

- Opening remarks: ECDC Director Andrea Ammon
- Danuta Skowronski, British Columbia Centre for Disease Control, Canada. ‘Immunological imprinting – the example of influenza’ (epidemiological perspective)
- Rory de Vries, Erasmus Medical Center, Rotterdam, The Netherlands. ‘Immune amnesia following infections with the measles virus’ (virological/immunological perspective)
- Jean-Daniel Lelièvre, Henri Mondor Universitary Hospital, Clinical Immunology & Infectious Diseases Department, Créteil, France. ‘The role of immunology supporting the design and adaptation of vaccination strategies’ (clinical and immunological perspective)

The online event is taking place on 19 November 2021 from 10:00- 11.30 CET. 

Participation is free of charge and interested experts are able to register via the ESCAIDE website. The deadline for registrations is 12 November 2021.

For more information about the event, please visit https://www.escaide.eu/en/news-events/eurosurveillance-25th-anniversary-seminar-epidemiology-and-immunology-observation.

To register, please visit https://www.escaide.eu/en/registration.

